# Current Practice and Perspectives on Subcutaneous Immunoglobulin Replacement Therapy in Patients with Primary Antibody Deficiency Among Specialized Nurses in Poland

**DOI:** 10.3390/nursrep14040238

**Published:** 2024-11-01

**Authors:** Dorota Mizera, Radosław Dziedzic, Anna Drynda, Aleksandra Matyja-Bednarczyk, Agnieszka Padjas, Magdalena Celińska-Löwenhoff, Bogdan Jakieła, Stanisława Bazan-Socha

**Affiliations:** 1 Jagiellonian University Medical College, Center for Innovative Medical Education, Medyczna 7, 30-688 Kraków, Poland; dorota.mizera@uj.edu.pl; 2 Jagiellonian University Medical College, Doctoral School of Medical and Health Sciences, Św. Łazarza 16, 31-530 Kraków, Poland; radoslaw.dziedzic@doctoral.uj.edu.pl; 3 Jagiellonian University Medical College, Students’ Scientific Group of Immune Diseases and Hypercoagulation, Jakubowskiego 2, 30-688 Kraków, Poland; anna.drynda@student.uj.edu.pl; 4 Jagiellonian University Medical College, Faculty of Medicine, Department of Internal Medicine, Jakubowskiego 2, 30-688 Kraków, Poland; aleksandra.matyja-bednarczyk@uj.edu.pl (A.M.-B.); agnieszka.padjas@uj.edu.pl (A.P.); magdalena.celinska-lowenhoff@uj.edu.pl (M.C.-L.); b.jakiela@uj.edu.pl (B.J.)

**Keywords:** immunodeficiency, immunoglobulin replacement therapy, nursing, survey

## Abstract

Background/Objectives: Inborn errors of immunity (IEI) encompass various congenital disorders, resulting in immunity defects and recurrent infections. Home-based subcutaneous immunoglobulin replacement therapy (scIgRT) is the best treatment option for those with primary antibody deficiency (PAD). However, the lack of standardized procedures in patient training remains a challenge. Our study investigates nurses’ practice and perspectives, aiming to identify areas for improvement in at-home scIgRT practice. Methods: We prepared a structured survey regarding scIgRT, including needle choice experience and perception of adverse events, and distributed it among qualified nurses involved in patient training and scIgRT supervising. Results: We included 56 nurses with a median age of 50 years. Among them, 67.9% represented adult care providers, while 32.1% supervised IgRT in children. Most respondents (83.9%) used the classic or assisted with hyaluronidase scIgRT preparations. Single-channel needles were administered most commonly (85.7%). The needle length was mostly chosen solely by a nurse (57.1%) or in cooperation with the patient (23.2%). Next, 9 mm and 12 mm needles were used most often (92.9% and 78.6%, respectively). As expected, the 6 mm needle was more frequently applied for children compared to adults (*n* = 16, 88.9% vs. *n* = 11, 28.9%, *p* < 0.001), while 12 mm was primarily used in adults (*n* = 35, 92.1% vs. *n* = 9, 50.0%, *p* < 0.001). Visual skin fold assessment was the basis for the needle selection (58.9%), followed by the injection site rule (26.8%) or a choice between two available needle types for thinner or thicker patients (25.0%). Results of this survey indicate that, according to nurses’ opinions presented in this survey, the needle length could be associated with local scIgRT adverse events, such as side needle leakage or local burning. Yet, it was likely unrelated to general adverse signs, such as headaches or dizziness. Most respondents (66.1%) indicated that, even if local adverse events occur, patients are reluctant to change scIgRT preparation or needle length. Most participants (69.6%) reported that the optimal administration technique needs to be discussed with the patient before and during scIgRT. Conclusions: This study sheds light on scIgRT practice in Poland, emphasizing deficiency in needle selection technique. Future research should focus on standardized training and advanced needle selection procedures on patient outcomes, investigating the correlation between needle strategies and adverse events, as well as the effectiveness of scIgRT.

## 1. Introduction

Inborn errors of immunity (IEI) constitute a heterogeneous group of congenital disorders related to several functional defects of the immune system. Among them, the most frequent are those referring to primary antibody deficiency (PAD), typified by an inability to provide an effective humoral response in case of variable B-cell functioning and antibody production defects [1]. As a result, PAD patients experience diverse clinical symptoms, including recurrent and severe infections, but also an increased risk of malignancy, allergy, and autoimmunity [2]. Therefore, IEI patients require complex approaches, including neoplastic vigilance, supportive care, infection prevention, and vaccinations [3]. However, for long-term outcomes of PAD patients, the most important is effective prolonged immunoglobulin replacement therapy (IgRT), which is prescribed in specialized healthcare centers providing comprehensive medical support for IEI patients [4,5].

IgRT may be administered intravenously in medical centers, requiring adequate peripheral venous access and experienced medical staff. An alternative is a subcutaneous administration in a home setting (scIgRT), which is beneficial for patients as it can be carried out at home, and it is currently widely used in most European countries [6]. In Poland, scIgRT has been approved for PAD by the National Health Fund since 2015. It is currently used by choice and is well accepted by patients and medical staff [7]. Furthermore, a recent paper by Windegger et al. [8] revealed that scIgRT in patients with hematological malignancies and concomitant hypoglobulinemia is also preferred over intravenous administration. Thus, one may speculate that the subcutaneous route of immunoglobulin substitution will also grow in importance in areas other than IEI. However, facilitating home-based scIgRT requires an individualized approach with patient education and proper training. Therefore, medical staff, usually specialized nurses, instruct the patients and their relatives about such issues as aseptic principles, the technique of subcutaneous injection depending on the type of needle used, the site of immunoglobulin administration, recognition of general and local adverse events, and procedures in case of their occurrence. Training also covers the operation of medical equipment and infusion pumps, manual administration of immunoglobulins without a pump, and handling medical waste generated during administration. Nevertheless, the scope of patient training has not been standardized in Poland, which may affect the frequency of side effects during infusion and influence patients’ comfort and anxiety regarding the procedure.

Since scIgRT in IEI is often lifelong [9], several challenges for patients and healthcare providers may be marked. Among them, the most important are managing local injection site reactions, optimizing needle selection to minimize discomfort, and addressing patient anxiety regarding the procedure [10]. Furthermore, the lack of standardized protocols for training healthcare professionals, particularly nurses in scIgRT, can result in variability in patient outcomes, emphasizing the need for improved educational tools and guidelines. Addressing these challenges is crucial for enhancing treatment efficacy and patient quality of life. From the patients’ perspective, common concerns include discomfort during needle insertion, anxiety related to the needle selection, and local adverse events such as pain, redness, or swelling at the injection site [11]. All of them may affect patient’s adherence and overall comfort. Moreover, many patients claim a need for more personalized care that considers their preferences and lifestyles and aligns with a patient-centered approach in clinical care [12].

Some earlier reports discussed the patient’s perspective regarding scIgRT [13,14]. However, to the best of our knowledge, studies focusing on the perspective of medical staff directly caring for patients with PAD are unavailable. In the current study, we sought to investigate contemporary practice and attitudes toward scIgRT techniques and patient training among nurses specialized in IgRT. We asked about scIgRT usage patterns, needle choice practices, adverse event perceptions, and reasons for changing immunoglobulin preparations or needle length. We believe such knowledge is necessary to identify areas for improvement in home-based scIgRT techniques, particularly since the responsibility for appropriate patient training and scIgRT management is currently attributed to the nursing team.

## 2. Materials and Methods

This study aimed to explore nurses’ practical experience and insights in scIgRT, focusing on improving procedural consistency in patient training, needle selection, and drug administration techniques. Our study had a cross-sectional design via a voluntary, anonymous, structured questionnaire prepared by the project authors, which was distributed directly to the nurses treating PAD patients. Participants for this study were recruited during the scientific conference “Immunology Nurses Forum 2022”, held on 16–17 September 2022, in Warsaw, under the theme “Smart Lifelong Care”. This conference was attended by representatives of nurses from most immunologic centers in Poland that provide IgRT under the National Health Fund reimbursement. This study targeted nurses who had only actively engaged in IgRT as follows: supervised at least ten PAD patients per year, for at least one year, and being responsive to patient training and drug administration. We asked about scIgRT practice, including needle choice and perception of adverse events. The survey had 5 sections with 19 questions (for details, see the Supplementary Materials File S1). The questionnaires were administered directly to the nurses (paper copy), who completed them in the presence of a trained team member responsible for ensuring proper data collection. Participants’ confidentiality and anonymity were ensured through coded identifiers, and informed consent was obtained, with participation being entirely voluntary. The team members who analyzed the results did not participate in data collection and did not know the responders’ names.

In the first part of the survey, respondents were prompted to indicate the sex, age, and province in which they work. The second section gathered information regarding the respondents’ experience and preference in scIgRT selection. Then, the respondents were asked to estimate the percentage of patients receiving subcutaneous preparations, including those with hyaluronidase, and types of needles used for subcutaneous immunoglobulin administration, including butterfly needles at a 45-degree angle and one to four-channel needles at a 90-degree angle. In the third part of the survey, participants provided insights into their approach to needle length selection for scIgRT. Respondents were asked to indicate the preferred needle length (6 mm, 9 mm, 12 mm, 14 mm, or 16 mm) for 90-degree injections, marking their frequency of use with options as “always”, “often”, “sometimes”, “never”, or “not known” for each one type of needle length. Then, the survey inquired about the decision-making process regarding the type and length of needle choice, including the following options: “nurse decision”, “physician decision”, “a joint decision involving the nurse, doctor, and patient”, “a joint decision between the doctor and nurse”, “a joint decision between the patient and nurse”, and “a joint decision between the doctor and patient”. Then, participants were asked to mark multiple relevant answers to explore needle length selection practice, including consideration based on patient weight, visual assessment of subcutaneous tissue thickness by pinching the skin fold, differentiation between needle lengths for thinner and thicker patients, patient selection, site-dependent choices, limited choice due to pharmacy-provided equipment, prevalence of butterfly needles inserted at 45 degrees, and an option for alternative methods.

Next, the fourth section of the survey was designed to investigate respondents’ perspectives on the potential association between subcutaneous immunoglobulin administration and general adverse signs. Participants were asked to assess whether general symptoms, such as headache, dizziness, vision problems, or musculoskeletal pain, could be attributed to using either too short or too long needles in scIgRT, including those with hyaluronidase. A similar inquiry was made regarding specific local symptoms related to the skin puncture and administration process. Respondents were prompted to indicate whether these symptoms influenced their decision to change scIgRT preparation or needle length and type.

Finally, we asked the respondents about additional aspects and follow-up practices, such as the manual push method and communication between medical staff and patients. Respondents were queried about the frequency of patient-initiated contacts related to unforeseen problems with scIgRT, categorized into “never”, “rarely”, “sometimes”, “often”, or “always in significant problems”. The survey also investigated whether healthcare professionals had encountered specific, more sophisticated 90-degree angle needle length selection procedures, such as using a caliper to measure skin fold thickness or employing ultrasound (USG) to measure subcutaneous tissue thickness.

During the research, we ensured the confidentiality and anonymity of participants. We did not offer any incentives, and participation was voluntary. The Bioethics Committee of the Jagiellonian University Medical College approved this research (No: 1072.6120.213.2021, on 29 September 2021).

The results were analyzed using SPSS software, version 29.0 (IBM Corporation, Armonk, NY, USA). Categorical variables were compared using the Chi^2^ test or exact Fisher test and presented as the number of subjects (*n*) with the percentage of total data (%). The normality of data distribution was evaluated using the Shapiro–Wilk test. All continuous variables were non-normally distributed and thus were presented in the manuscript as a median with Q1–Q3 quartile range. Results were considered statistically significant in cases of *p*-value less than 0.05.

## 3. Results

### 3.1. Respondent Characteristics

In general, 56 nurses, all women, answered the questions. They represented specialized in PAD therapy centers from the following provinces of Poland: Lesser Poland (*n* = 2, 3.6%), Subcarpathian (*n* = 4, 7.1%), Silesian (*n* = 3, 5.4%), Masovian (*n* = 7, 12.5%), Pomeranian (*n* = 9, 16.1%), Greater Poland (*n* = 7, 12.5%), Swietokrzyskie (*n* = 3, 5.4%), Lower Silesian (*n* = 7, 12.5%), Warmian–Masurian (*n* = 3, 5.4%), Lodz (*n* = 7, 12.5%), and Podlasie (*n* = 4, 7.1%). The median age of the respondents was 50 years, with the largest group (*n* = 39, 69.6%) aged 46–61. 64.3% of respondents (*n* = 36), which indicated that they had been involved in the IgRT for at least 5 years; 16.1% (*n* = 9) have one year of experience, while the remaining 19.6% (*n* = 11) treated PAD patients for 2 to 4 years. Furthermore, respondents more often care for adult patients (*n* = 38, 67.9%) than children (*n* = 18, 32.1%). Detailed information on the demographic characteristics of respondents is provided in Table 1.

### 3.2. Patterns of Subcutaneous Immunoglobulin Usage and Decision-Making Processes

Most respondents (*n* = 50, 83.3%) used both types of subcutaneous immunoglobulins, i.e., classic ones, and were assisted by hyaluronidase. The most often used were single-channel needles (*n* = 48, 85.7%), followed by butterfly needles inserted at a 45-degree angle (*n* = 20, 35.7%) and double-channel needles (*n* = 19, 33.9%) injected at an angle of 90 degrees. The decision regarding needle length was usually made solely by nurses (*n* = 32, 57.1%), followed by a collective decision of the patient and nurse (*n* = 13, 23.2%) or the physician and nurse (*n* = 9, 16.1%). One respondent claimed that only one needle type is available at the hospital pharmacy onsite, and another one said that it is a joint decision between the doctor, nurse, and patient. We summarized information about answers on IgRT by respondents in Table 2.

### 3.3. Variability in Needle Length Usage Among Respondents

The most often used was 9 mm needle (*n* = 52, 92.9%), both in children’s care (*n* = 18, 34.6%) and in adult’s care (*n* = 34, 65.4%). Furthermore, almost half of the respondents (*n* = 27, 48.2%) chose a 6 mm long needle, mostly in children than in adults (*n* = 16, 88.9% vs. *n* = 11, 28.9%, *p* < 0.001, the exact Fisher test). The 12 mm needle was used by 78.6% of respondents (*n* = 44), mainly in adults than in children (*n* = 35, 92.1% vs. *n* = 9, 50.0%, *p* < 0.001, the exact Fisher test). One of the longest available needles, 14 mm, was applied by 16.1% of practitioners (*n* = 9) and only in adults (*n* = 9, 100.0%). No respondents used 4 mm or 16 mm needle sizes. We have presented detailed information in Table 3.

### 3.4. A Comparison of Rules in Immunoglobulin Administration in Children and Adult Patients

The most common rule for choosing the needle length was visually assessing the skin fold, as reported by more than half of the respondents (*n* = 33, 58.9%), more often in pediatric than in adult care centers (*p* = 0.048). Other frequent methods for choosing a needle length were based on the injection site, with longer needles for the abdomen and shorter for the thigh or arm (*n* = 15, 26.8%). In some centers, only two types of needles were available: the short for thinner and the long for thicker ones (*n* = 14, 25.0%). Detailed information is presented in Table 4.

Interestingly, some centers used the method based on the patient’s weight: the greater the weight, the longer the needle, and the lower the weight, the shorter the needle (*n* = 12, 21.4%), but without strict recommendations. In one center, only one needle size was available. None of the responders used a skinfold caliper or USG in needle selection.

### 3.5. Perceptions of Needle Length and Adverse Events Among Respondents

Most surveyed nurses claimed that, in their opinion, the occurrence of general adverse events in patients is unrelated to the length of the needle used for scIgRT. Detailed information on this topic is provided in Table 5. For example, 94.6% of respondents did not connect vision problems, tinnitus, or diarrhea with needle length. Interestingly, almost one-fifth (*n* = 11, 19.6%) believed that too-long needles might be associated with musculoskeletal pain.

In turn, opinions on local adverse effects varied. More than half of the respondents did not link the needle length with skin puncture difficulty (57.1%), pain during needle insertion (55.4%), and a warm feeling in the needle insertion area (53.6%). On the other hand, the majority claimed that the short needle might be related to fluid leakage from the needle (85.7%), bubbles near the inserted needle (60.7%), and burning at the administration site during immunoglobulin administration (57.1%). The most common local symptoms associated with too-long needles might be pain during immunoglobulin administration (28.6%), bloody discharge after needle removal (32.1%), and pain during needle insertion (28.6%). Detailed information is shown in Table 6.

### 3.6. Reasons for Changing Immunoglobulin Preparations

In the next part of the survey, nurses were asked whether patient symptoms prompted a change in the immunoglobulin preparation or needle selection. The majority (*n* = 37, 66.1%) indicated no need for such changes. However, one-third of respondents (*n* = 19, 33.9%) acknowledged replacement due to various reasons, such as significant local itchy reactions, pain during needle insertion, redness at the injection site, swelling during immunoglobulin administration, headaches, nausea, vomiting, sub-febrile states, flu-like symptoms, or other post-transfusion signs. The most frequently cited reasons for the change were swelling at the immunoglobulin administration site (*n* = 6), followed by pain during administration (*n* = 5), and severe itching and erythema at the application site (*n* = 3).

### 3.7. Home Immunoglobulin Administration and Communication Between Patients and Medical Staff

Manual immunoglobulin administration without needing an infusion pump was not widely known and used among respondents. Only about one-fifth (*n* = 11, 19.6%) used it in clinical practice, but the others (*n* = 45, 80.4%) never tried or heard about such a procedure.

Next, the vast majority (*n* = 39, 69.6%) of respondents claimed that patients can always discuss problems regarding infusion and/or adverse events that occurred during administration with them. Surprisingly, six respondents (10.7%) stated they rarely or never discuss such issues with patients.

Regarding the frequency of patients contacting medical staff for issues other than adverse events of scIgRT, 96.4% of respondents reported such interactions. Specifically, 33.9% (*n* = 19) were always, 21.4% (*n* = 12) were often, 16.1% (*n* = 9) were sometimes, and 25.0% (*n* = 14) were rarely contacted, and only 3.6% (*n* = 2) of respondents claimed that patients never contacted them.

## 4. Discussion

To our knowledge, this is the first study to provide insights into experience and perspectives on scIgRT practice among nurses in Poland. It sheds light on crucial aspects of the home scIgRT by revealing current patterns and identifying areas for care improvement. The findings, representing a novel contribution to the field, underscore the need for comprehensive discussions around better practice regarding at-home scIgRT, particularly from the nurses’ point of view. Since patients on scIgRT report a better quality of life compared to intravenous IgRT [15], this topic is highly relevant for healthcare.

Essential for at-home immunoglobulin therapy is proper patient education, which encompasses aseptic handling, immunoglobulin transport and storage, adverse reactions, procedures in case of their occurrence, and infusion pump operation. All those training aspects in Poland and other European countries rely on nurse teams. However, in Poland, there are no recommendations on what should be discussed during such training; thus, the program depends on the experience and skills of the nurse. The care of PAD patients receiving IgRT is crucial in implementing a therapeutic plan, monitoring treatment, and evaluating the patient’s response. The nurse’s insight and attitude are the most significant in all those aspects [16]. It is worth highlighting that the exchange of experiences, insights, and the implementation of innovations in scIgG therapy takes place mainly at national but also international conferences for nurses, some of whom are members of the international organization, the “International Nursing Group for Immunodeficiencies” (INGID).

Half of the respondents claimed that technical issues regarding scIgRT, particularly needle choice and site of administration, depend only on the nurse’s decision. In another one-fourth, this decision is made through cooperation between the nurse and patient. It indicates the main nurse’s responsibility regarding scIgRT, again pointing to the need to prepare clear recommendations and better tools to support decisions, e.g., optimal needle choice. Currently, it is based only on the experience of the nurse.

About one-third of patients receiving intravenous IgRT experience adverse events, which are mostly mild and easy to manage [17]. In patients on scIgRT, side effects are less frequent, but they also might happen. Although our respondents claimed that onsite adverse events are related to the administration technique and also needle length, the majority of them did not see reasons for immunoglobulin or needle length adjustment. It indicates that nurses are hesitant to implement such changes, and possible physician involvement in that team would change the approach. A well-functioning therapeutic team can quickly identify concerning behaviors and implement modifications to achieve desired success and treatment satisfaction. Furthermore, no well-designed studies analyzed the frequency and severity of local adverse reactions during scIgRT, depending on needle type and length. We plan to address that issue in our future research.

Finally, all but six of our respondents claimed that patients can always contact them regarding any medical problems, not only referring to scIgRT. That is a critical aspect of the patient’s well-being. Patients undergoing home therapy must be committed to it and should not be coerced. Trust in the medical staff provides a significant sense of security in the patient’s and their family’s actions. Therapeutic teams involving physicians, nurses, pharmacists, patients, and their families should collaborate to maximize treatment effectiveness and safety. In general, health-related quality of life in PAD patients is notably lower than in healthy populations. Thus, proper and safe home-based scIgRT seems critical in improving overall well-being [18]. At the same time, better communication with medical staff could increase trust and improve the quality of life of PAD patients and their families.

The present study has some limitations. Firstly, the sample consists of a limited number of female respondents; however, we included only immunologic nurses experienced and specialized in IgRT who supervised at least ten PAD patients per year for at least one year. Secondly, potential response bias may exist due to the subjective nature of participants’ responses, rooted in their individual experiences. However, we aimed to investigate a subjective approach of nurses to the at-home scIgRT, and to the best of our knowledge, it is the first study on this issue. One limitation of this study is the potential for selection bias due to convenience sampling and using our own, not-validated questionnaire. We also did not ask whether the nurse had undergone formalized training in scIgRT techniques. Nevertheless, we know that every nurse undergoes both practical and theoretical training before administering subcutaneous immunoglobulins and subsequently teaching patients. Certification of this training is not mandatory before starting care for patients with PAD in Poland.

## 5. Conclusions

In the present study, we highlight the lack of recommendations for optimal at-home scIgRT care in PAD patients. Our survey shows a need for unified nurses’ and patients’ training, better needle selection procedures, and close cooperation between the doctor, nurse, and patient. Further studies on that issue could improve the quality of at-home scIgRT care.

## Figures and Tables

**Table 1:** A summary of demographic characteristics of respondents.

Characteristics	Respondents *n* = 56
Age, years	50.0 (41.3–55.3)
Female sex, *n* (%)	56 (100.0%)
Time in practice in IgRT, years	5.0 (3.0–9.0)
Time in practice in IgRT with needles inserted at 90 degrees, years	4.0 (3.0–5.0)
Type of patients that medical staff care for, *n* (%)	Adults: 38 (67.9%)
	Children: 18 (32.1%)

Categorical variables are presented as numbers with percentages, and the continuous variable (age) as a median with Q1–Q3 range. IgRT—immunoglobulin replacement therapy.

**Table 2:** A summary of answers about subcutaneous immunoglobulin replacement therapy practice.

Characteristics	Parameter	Respondents *n* = 56
Type of immunoglobulins used in immunodeficiencies patients, *n* (%)	only classic subcutaneous immunoglobulins	4 (7.1%)
	only subcutaneous immunoglobulins assisted by hyaluronidase	5 (8.9%)
	both types of subcutaneous immunoglobulins	47 (83.9%)
Type of needle, *n* (%)	usage of single-channel needles inserted at 90 degrees	48 (85.7%)
	usage of double-channel needles inserted at 90 degrees	19 (33.9%)
	usage of triple-channel needles inserted at 90 degrees	5 (8.9%)
	usage of butterfly needles inserted at 45 degrees	20 (35.7%)
Decision-making authority for subcutaneous immunoglobulin needle selection, *n* (%)	nurse	32 (57.1%)
	joint decision of the patient and nurse	13 (23.2%)
	joint decision of the doctor and nurse	9 (16.1%)
	joint decision of doctor, nurse, and patient	1 (1.8%)
	single type ordered by the hospital pharmacy	1 (1.8%)

Categorical variables are presented as numbers with percentages.

**Table 3:** A summary of needle length selection preference (injected at an angle of 90 degrees) for subcutaneous immunoglobulin administration.

Needle Length	Frequency of Specific Needle Length Usage by Respondents				
	Always	Often	Sometimes	Never	Not Known
4 mm, *n* (%)	0 (0.0%)	0 (0.0%)	0 (0.0%)	56 (100.0%)	0 (0.0%)
6 mm, *n* (%)	3 (5.4%)	12 (21.4%)	12 (21.4%)	29 (51.8%)	0 (0.0%)
9 mm, *n* (%)	10 (17.9%)	26 (46.4%)	16 (28.6%)	4 (7.1%)	0 (0.0%)
12 mm, *n* (%)	9 (16.1%)	29 (51.8%)	6 (10.7%)	11 (19.6%)	0 (0.0%)
14 mm, *n* (%)	0 (0.0%)	1 (1.8%)	6 (10.7%)	48 (85.7%)	1 (1.8%)
16 mm, *n* (%)	0 (0.0%)	0 (0.0%)	0 (0.0%)	56 (100.0%)	0 (0.0%)

Categorical variables are presented as numbers with percentages.

**Table 4:** A comparison in rules of immunoglobulin administration in children and adult patients.

Rule of Immunoglobulin Administration	All Respondents *n* = 56	Adult Care Patients *n* = 38	Children Care Patients *n* = 18	*p*-Value Adults vs. Children Care
I pinch a skin fold and visually assess the thickness of subcutaneous tissue. The thicker the fold, the longer the needle. *n* (%).	33 (58.9%)	19 (50.0%)	14 (77.8%)	0.048
I adjust the needle length based on the administration site (e.g., longer for the abdomen, shorter for the thigh or arm). *n* (%).	15 (26.8%)	10 (26.3%)	5 (27.8%)	1.00
I have two types of needles available, and I use shorter needles for leaner patients and longer needles for more robust patients. *n* (%).	14 (25.0%)	12 (31.6%)	2 (11.1%)	0.19
Based on the patient’s weight, the smaller the weight, the shorter the needle, and the larger the weight, the longer the needle. *n* (%).	12 (21.4%)	6 (15.8%)	6 (33.3%)	0.17
I have only one size of needles available, and I use them uniformly. *n* (%).	7 (12.5%)	5 (13.2%)	2 (11.1%)	1.00
Most patients administer immunoglobulins using butterfly needles at a 45-degree angle. *n* (%).	3 (5.4%)	1 (2.6%)	2 (11.1%)	0.24
I do not influence needle length selection because the pharmacy provides the equipment and medications. *n* (%).	2 (3.6%)	2 (5.3%)	0 (0.0%)	1.00
Patients choose the needle length that suits them best. *n* (%).	2 (3.6%)	2 (5.3%)	0 (0.0%)	1.00
The nurse handles the needle length selection. *n* (%).	0 (0.0%)	0 (0.0%)	0 (0.0%)	NA

Categorical variables are presented as numbers with percentages. Statistically significant differences are marked in bold. NA—not applicable.

**Table 5:** A summary of survey results on the subjective perception of general adverse events and possible reasons for their occurrence during immunoglobulin administration.

General Adverse Events	Possible Explanation for the Occurrence of Adverse Events			
	Too-Long Needle	Too-Short Needle	Unrelated to Needle Length	Not Known
Headache, *n* (%)	5 (8.9%)	0 (0.0%)	50 (89.3%)	1 (1.8%)
Dizziness, *n* (%)	4 (7.1%)	0 (0.0%)	51 (91.1%)	1 (1.8%)
Vision problems, *n* (%)	1 (1.8%)	0 (0.0%)	53 (94.6%)	2 (3.6%)
Tinnitus, *n* (%)	1 (1.8%)	0 (0.0%)	53 (94.6%)	2 (3.6%)
Musculoskeletal pain, *n* (%)	11 (19.6%)	1 (1.8%)	44 (78.6%)	0 (0.0%)
Increased body temperature, *n* (%)	3 (5.4%)	6 (10.7%)	45 (80.4%)	3 (5.4%)
Decreased blood pressure, *n* (%)	6 (10.7%)	2 (3.6%)	48 (85.7%)	2 (3.6%)
Rapid heartbeat, *n* (%)	7 (12.5%)	2 (3.6%)	46 (82.1%)	3 (5.4%)
Back pain, *n* (%)	2 (3.6%)	0 (0.0%)	52 (92.9%)	2 (3.6%)
Chest pain, *n* (%)	2 (3.6%)	1 (1.8%)	52 (92.9%)	1 (1.8%)
Cough, *n* (%)	2 (3.6%)	1 (1.8%)	51 (91.1%)	2 (3.6%)
Dyspnoea, *n* (%)	3 (5.4%)	1 (1.8%)	50 (89.3%)	1 (1.8%)
Nausea, *n* (%)	2 (3.6%)	0 (0.0%)	52 (92.9%)	2 (3.6%)
Syncope, *n* (%)	5 (8.9%)	0 (0.0%)	50 (89.3%)	1 (1.8%)
Vomiting, *n* (%)	2 (3.6%)	0 (0.0%)	52 (92.9%)	2 (3.6%)
Diarrhea, *n* (%)	1 (1.8%)	0 (0.0%)	53 (94.6%)	2 (3.6%)

Data reflect the nurses’ opinions based on their professional experience. Categorical variables are presented as numbers with percentages.

**Table 6:** A summary of survey results on the subjective perception of the association between local adverse effects and needle length.

Local Adverse Effect	Possible Explanation for the Occurrence of Adverse Effect			
	Too-Long Needle	Too-Short Needle	Unrelated to Needle Length	Not Known
Skin puncture difficulty, *n* (%)	0 (0.0%)	21 (37.5%)	32 (57.1%)	3 (5.4%)
Pain during needle insertion, *n* (%)	16 (28.6%)	9 (16.1%)	31 (55.4%)	1 (1.8%)
Pain during immunoglobulin administration, *n* (%)	17 (30.4%)	23 (41.1%)	18 (32.1%)	0 (0.0%)
Swelling after immunoglobulin administration, *n* (%)	3 (5.4%)	26 (46.4%)	24 (42.9%)	3 (5.4%)
Significant warming in the needle insertion area, *n* (%)	3 (5.4%)	22 (39.3%)	30 (53.6%)	2 (3.6%)
Redness at the administration site, *n* (%)	3 (5.4%)	26 (46.4%)	26 (46.4%)	2 (3.6%)
Burning at the administration site, *n* (%)	9 (16.1%)	32 (57.1%)	19 (33.9%)	2 (3.6%)
Bubble near the inserted needle, *n* (%)	1 (1.8%)	34 (60.7%)	18 (32.1%)	3 (5.4%)
White halo in the proximity of the inserted needle, *n* (%)	3 (5.4%)	27 (48.2%)	21 (37.5%)	5 (8.9%)
Erythema, *n* (%)	4 (7.1%)	27 (48.2%)	23 (41.1%)	3 (5.4%)
Fluid leakage from the needle, *n* (%)	0 (0.0%)	48 (85.7%)	6 (10.7%)	2 (3.6%)
Bloody discharge after needle removal, *n* (%)	18 (32.1%)	12 (21.4%)	22 (39.3%)	4 (7.1%)

Data reflect the nurses’ opinions based on their professional experience. Categorical variables are presented as numbers with percentages.

## Data Availability

The data presented in this study are available on reasonable request from the corresponding author.

## Supplementary Materials

The following supporting information can be downloaded at: https://www.mdpi.com/article/10.3390/nursrep14040238/s1, File S1: current perspectives on subcutaneous immunoglobulin replacement therapy in primary antibody deficiency patients among specialized nurses in Poland—survey (version translated into English).

## Public Involvement Statement

No public involvement in any aspect of this research.

## Guidelines and Standards Statement

This manuscript was drafted against the STROBE guidelines.
