# Supplementary material for: Current Practice and Perspectives on Subcutaneous Immunoglobulin Replacement Therapy in Patients with Primary Antibody Deficiency Among Specialized Nurses in Poland
Source: Nurs Rep. 2024 Nov 1;14(4):3280–90. doi: 10.3390/nursrep14040238 (PMC11587484; doi:10.3390/nursrep14040238)
Supplement: Supplementary file 1 [file nursrep-14-00238-s001.zip › nursrep-3163759-supplementary.pdf]

Supplementary materials

Current perspectives on subcutaneous immunoglobulin replacement therapy in primary antibody deficiency patients among specialized nurses in Poland – survey

We invite you to fill out the presenting form addressed to medical staff dealing with the treatment of primary antibody deficiency patients. The study aims to explore the perspectives of medical staff on immunoglobulin replacement therapy to identify areas for care improvement.

The following survey consisted of 5 sections with 19 questions in total. Please respond to all questions. In each question, please select one answer unless otherwise specified.

Thank you very much for participating in this study!

Section 1: Personal information and professional details

1. Please choose your sex, and mark only one square.
    - □ male
    - □ female

2. Please write your age in years.
    - _______

3. Please write a province, where you work with patients with immunodeficiencies.
    - ____

Section 2: Experience with subcutaneous immunoglobulins and patients treatment

4. How long have you been administering subcutaneous immunoglobulins (including those using hyaluronidase)? Please, mark only one square.
    - □ less than a year
    - □ 1 year
    - □ 2 years
    - □ 3 years
    - □ more, then please specify how many years: __________
5. How long have subcutaneous immunoglobulins (including those using hyaluronidase) with 90-degree angle needles been practiced in your experience? Please, mark only one square.
    - □ less than a year
    - □ 1 year
    - □ 2 years
    - □ 3 years
    - □ more, then please specify how many years? __________
6. Please specify the patients under your care. Please, mark only one square.
    - □ adults above 18 years
    - □ children up to 18 years
7. What type of subcutaneous immunoglobulins is administered in your Immune Deficiency Treatment Center? Please, mark only one square.
    - □ only subcutaneous immunoglobulins
    - □ only subcutaneous immunoglobulins with hyaluronidase
    - □ both types of immunoglobulins are administered
8. What type of needles for subcutaneous immunoglobulin administration (including those with hyaluronidase) is available in your Immune Deficiency Treatment Center? You can choose multiple answers by marking "X".
    - □ butterfly needles – inserted at a 45-degree angle
    - □ 1-channel needles inserted at a 90-degree angle
    - □ 2-channel needles inserted at a 90-degree angle
    - □ 3-channel needles inserted at a 90-degree angle
    - □ 4-channel needles inserted at a 90-degree angle

Section 3: Needle length selection principles

9. What length of 90-degree angle needles for subcutaneous immunoglobulin administration (including those with hyaluronidase) do you usually choose for your patients? Please mark "X" in each line.

	always	often	sometimes	never	not known
6 mm					
9 mm					
12 mm					
14 mm					
16 mm					

10. In your Immune Deficiency Treatment Center, who decides on the type and length of the needle used for subcutaneous immunoglobulin administration (including those with hyaluronidase)? Please, mark only one square.
    - □ joint decision of the nurse, doctor, and patient
    - □ joint decision of the doctor and nurse
    - □ joint decision of the patient and nurse
    - □ joint decision of the doctor and patient
    - □ there is no decision made, explain why: ___________________________

11. What principle do you follow in selecting the length of 90-degree angle needles for subcutaneous immunoglobulin administration (including those with hyaluronidase) for patients? Please mark "X", you can choose multiple answers.
    - □ Based on the patient's weight – shorter needle for lower weight, longer needle for higher weight
    - □ Pinch the skin fold and visually assess the thickness of subcutaneous tissue – thicker fold, longer needle
    - □ I have only one size of needles available, and I use that size
    - □ I have two types of needles available, and I use shorter needles for slimmer patients and longer needles for sturdier ones
    - □ Patients choose the needle length that suits them □ Needle length depends on the administration site (e.g., longer for abdomen, shorter for thigh or arm)
    - □ I do not have an influence on needle length selection, as the pharmacy provides equipment and medications
    - □ Most patients administer immunoglobulins with butterfly needles inserted at a 45-degree angle
    - □ Other method. Please describe it. _______________________________________

Section 4: Symptoms and changes in subcutaneous immunoglobulin treatment

12. Do you think the listed general symptoms may be caused by using either too short or too long needles for subcutaneous immunoglobulin administration (including those with hyaluronidase)? Please mark "X" in each line.

	Too short needle	Too long needle	No connection with a needle length
Headache			
Dizziness			
Vision problems			
Tinnitus			
Musculoskeletal pain			
Increased body temperature			
Decreased blood pressure			
Increased heart rate			
Back pain			
Chest pain			
Cough			
Dyspnoea			
Nausea			
Vomiting			
Fainting			
Diarrhea			

13. Do you think the listed general symptoms may be caused by using either too short or too long needles for subcutaneous immunoglobulin administration (including those with hyaluronidase)? Please mark "X" in each line.

	Too short needle	Too long needle	No connection with a needle length
Problem with skin puncture			
Pain during needle insertion			
Pain during immunoglobulin administration			
Swelling after immunoglobulin administration			
Significant warming around the needle insertion site			
Redness at the site of immunoglobulin administration			
Burning at the administration site			
Bubble near the inserted needle			
White halo near the inserted needle			
Leakage of fluid from under the needle			
Bloody leakage after needle removal			

14. Has any of the symptoms listed in points 12 and 13 been a reason to change subcutaneous immunoglobulin preparations (including those with hyaluronidase) for patients in your Immune Deficiency Treatment Center? Please, mark only one square and if yes, please explain.
    - □ no
    - □ yes, which one(s)? ____

Section 5: Additional aspects and follow-up

15. In your Immune Deficiency Treatment Center, do patients use the manual push method for subcutaneous immunoglobulin administration without using a pump? Please, mark only one square.
    - □ yes
    - □ sometimes
    - □ no
    - □ I do not know
16. How often during follow-up visits in the Immune Deficiency Treatment Center do you talk to the patient about the administered infusions? Please, mark only one square.
    - □ never
    - □ rarely
    - □ sometimes
    - □ always
17. How often do patients contact you due to unforeseen problems with subcutaneous immunoglobulin administration? Please, mark only one square.
    - □ never
    - □ rarely
    - □ sometimes
    - □ often
    - □ always when they have a significant problem
18. Have you ever encountered the use of a 90-degree angle needle length selection for subcutaneous immunoglobulin administration (including those with hyaluronidase) by measuring the thickness of the skin fold using a caliper? Please, mark only one square.
    - □ no
    - □ yes
19. Have you ever encountered the use of a 90-degree angle needle length selection for subcutaneous immunoglobulin administration (including those with hyaluronidase) by measuring the thickness of subcutaneous tissue using ultrasound (USG)? Please, mark only one square.
    - □ no
    - □ yes

Thank you for completing the survey!
